# Vortex beam manipulation through a tunable plasma-ferrite metamaterial

**DOI:** 10.1038/s41598-021-95693-1

**Published:** 2021-08-06

**Authors:** Davod Nobahar, Sirous Khorram, João D. Rodrigues

**Affiliations:** 1 Faculty of physics, University of Tabriz, Tabriz, 51666-16471 Iran; 2 Research Institute for Applied Physics and Astronomy, University of Tabriz, Tabriz, 51666-16471 Iran; 3 Blackett Laboratory, Physics Department, Imperial College London, Prince Consort Road, London, SW7 2AZ UK

**Keywords:** Materials science, Optics and photonics

## Abstract

This paper is devoted to the study of vortex beam transmission from an adjustable magnetized plasma-ferrite structure with negative refraction index. We use the angular spectral expansion technique together with the $4\times 4$ matrix method to find out the transmitted intensity and phase profiles of incoming Laguerre-Gaussian beam. Based on numerical analysis we demonstrate that high transparency and large amount of Faraday rotation in the proximity of resonance frequency region, reverse rotation of spiral wave front, and side-band modes generation during propagation are the remarkable features of our proposed structure. These controllable properties of plasma-ferrite metamaterials via external static magnetic field and other structure parameters provide novel facilities for manipulating intensity and phase profiles of vortex radiation in transmission through the material. It is expected that the results of this work will be beneficial to develop active magneto-optical devices, orbital angular momentum based applications, and wavefront engineering.

## Introduction

In the last years, new advancements in artificial sub-wavelength materials have led to the emergence of negative refraction index structures which have simultaneously negative magnetic permeability and electric permittivity in some frequency domains^1–3^. Such engineered composites, named metamaterials, have brought about promising platforms and great opportunities to make salient developments in various area as optical imaging^4,5^, and photonic engineering^6,7^. It is well established that metamaterials can be designed in diverse geometrical and physical configurations which may lead to complications in some cases. In this regard, Podolskiy et al.^8^ offered a simpler way to acquire negative refraction index without simultaneous creation of magnetic and electric resonances in a one material. This was the first step to use periodic stratified structures as a metamaterial in which alternative layers of two ordinary materials produce a left handed medium within a certain frequency range^9^.

Stratified metamaterials owning to the multi reflection and transmission characteristics have provided effective media in order to modify amplitude, phase, location, and polarization of incoming electromagnetic waves^10–12^. Especially, employing external electric or magnetic fields over such inhomogeneous media enables a higher degree of control over the radiation field through anisotropy-related phenomena such as optical birefringence^13,14^. In recent years, indisputable prevalence of metamaterial technologies has overshadowed the plasma applications, and aroused a burgeoning interest in exploring optical and magneto-optical properties of plasma metamaterials^15–17^. In comparison with ordinary metamaterials, promptly control of plasma permittivity function through external parameters makes it a highly adequate for fabricating tunable metastructures. Furthermore, due to the variation of internal parameters of plasma such as electron density, it can cover a wide band of electromagnetic frequency^18^. Similar to the plasma features, the magnetic permeability function of a ferrite layer is greatly dependent to the static external magnetic field^19,20^. Inspired by these unique functionalities, here we propose an adjustable stratified metamaterial constructed from periodic plasma and ferrite layers to manipulate incoming microwave vortex beam (VB), and demonstrate what happens to the intensity distribution, phase map, polarization direction, and orbital angular momentum (OAM) of a VB while normally passes through a plasma-ferrite metamaterial (PFMM) layout.

About 29 years ago, the concept of twisted beams carrying OAM was proposed by Allen and his co-workers^21^. They pointed out that besides spin angular momentum, electromagnetic waves can also carry OAM when their Poynting vector rotates around the propagation axis. It is well proved that VBs manifest themselves through hollow annular pattern with a phase singularity at the center of their helical wavefront^22–24^. These specific features of VBs have offered new degrees of freedom to make further advances in high resolution imaging and microscopy^25^, optical trapping^26^, data transmission and wireless communication^27^, quantum technology^28^, and so on. As a pivotal role in VBs communication, it can be seen that in the last two decades much effort has been devoted to study the interaction of Laguerre-Gaussian (LG) and Bessel beams with multilayer structures and other complex media^29–32^. More remarkably, applying programmable metasurfaces to generate and reshape OAM modes has conveyed research in the field of data encoding to new plateau^33–36^. To this end, several theoretical approaches have been employed to unravel the mechanism of VBs propagation in different types of media^37,38^. Since the magnetized PFMM behaves as an anisotropic uniaxial medium, in this paper we utilize angular spectral expansion (ASE) technique accompanied by $4\times 4$ matrix method. This matrix formalism^39^ is a powerful approach to solve the problems connected to the anisotropic multilayer media, and handily governs the intricacy of various modes of polarization coupling at the anisotropic layer interface^40–42^.

The rest part of this paper is organized as follows. At first, we adopt the ASE technique to transform a LG beam into superposition of plane waves, and define the effective negative refraction index of PFMM, and find the transmission coefficient of VB via the $4\times 4$ matrix formalism. Then, we numerically investigate the output beam profiles and describe a variety of magneto-optical effects. Finally, the results of this work are summarized and conclusion are stated.

## Summary of theoretical model

**Figure 1:**
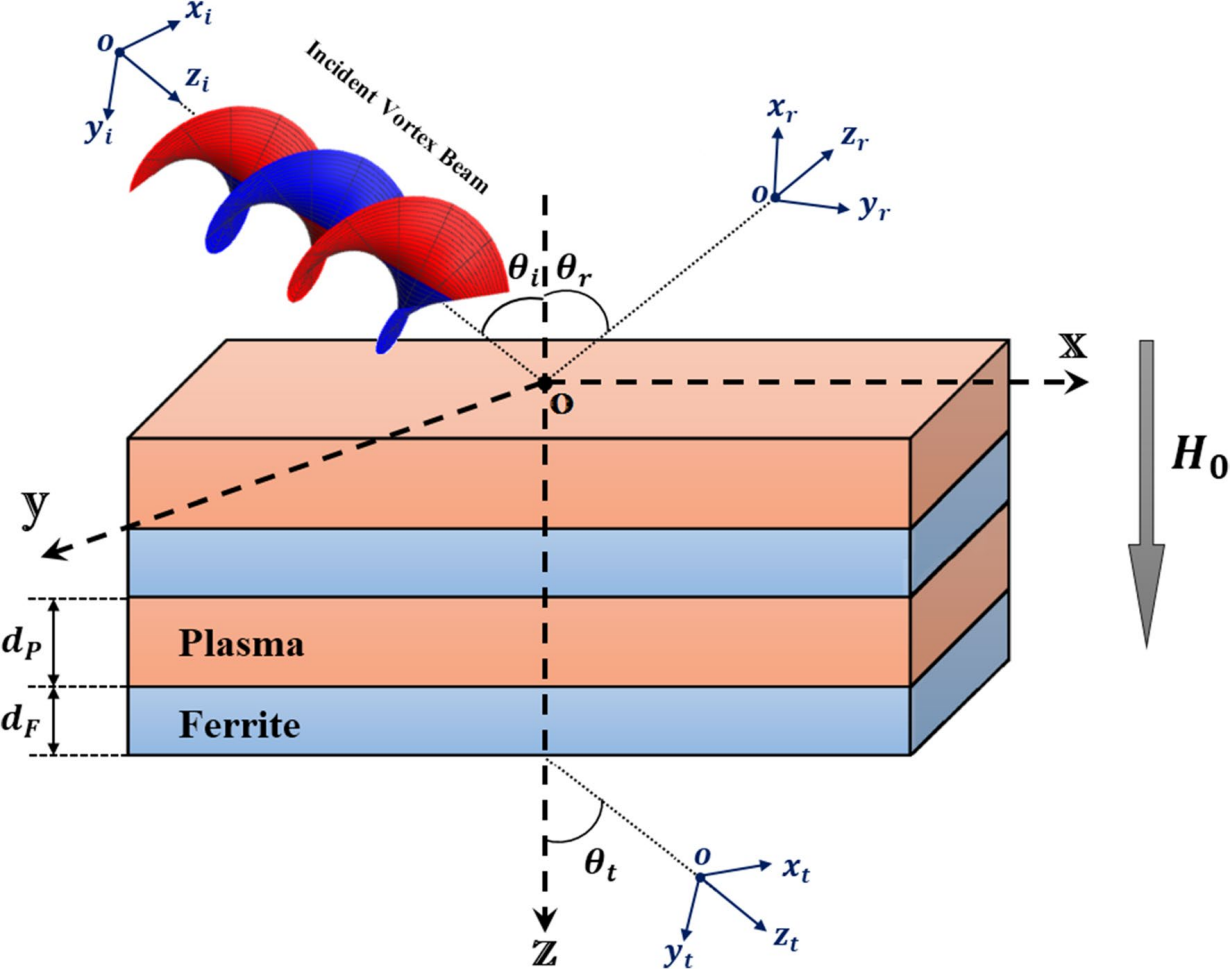
Schematic view of a VB interaction with a longitudinally magnetized PFMM.

The configuration of the stratified metamaterial under investigation is schematically illustrated in Fig. 1. An incident VB on the top surface of the PFMM with an incidence angle $\theta _{i}$ is partially transmitted and reflected with the angles $\theta _{t}$ and $\theta _{r}$, respectively. We assume that *oxyz* is a global coordinate system, $ox_{\alpha }y_{\alpha }z_{\alpha }$ $(\alpha \in \{i,r,t\})$ are the local coordinates attached to the incident and output beams, $d_{F}$ is the thickness of the ferrite layer, and $d_{P}$ is the thickness of the plasma layer. Furthermore, in our model the periodic PFMM is affected by a static extenal magnetic field $\vec {H}_{0}$ aligned along the ${\hat{z}}$-direction. This imposed magnetic field induces an anisotropy in the ferrite and plasma layers, and causes the plasma permittivity and ferrite permeability to be a tensor instead of a scalar. In this problem, we utilize from a linearly polarized LG beam as an incident VB. The electric field of such VB in the cylindrical coordinate system is expressed by

1 $$\begin{aligned} E_{i}(r_{i},\phi _{i}, z_{i})= \frac{1}{R_{0}} \sqrt{\frac{2 p!}{\pi (p+ |\ell |)!}}(\frac{\sqrt{2} r_{i}}{R_{0}})^{|\ell |} \exp [\frac{-r_{i}^{2}}{R_{0}^{2}}] L_{p}^{\ell }(\frac{2 r_{i}^{2}}{R_{0}^{2}}) exp [-i(\ell \phi _{i}+ \omega t)], \end{aligned}$$

where, $R_{0}$ is the beam waist radius, *p* is the radial mode number or hyperbolic momentum charge, $\ell$ is the angular mode number or topological charge, $L_{p}^{\ell }$ is the Laguerre polynomial, and $\omega$ is the frequency of incident VB. It is known that the VB calculations are accompanied by some complexity. This difficulties can be avoided in the frame of ASE^43,44^, in which the VB is decomposed into infinite plane waves in the spectral domain as follows

2 $$\begin{aligned} {\tilde{E}}_{i}(k_{i}, \varphi _{i})=\frac{1}{2\pi }\int _{0}^{\infty }\int _{0}^{2\pi } E_{i}(r_{i},\phi _{i}, z_{i})\exp [-i k_{i}r_{i}\cos (\phi _{i}-\varphi _{i})] r_{i}dr_{i}d\phi _{i}. \end{aligned}$$

Here, $(k_{i},\varphi _{i})$ indicates the incident spectrum space. In this case, the transmitted electric field in the output plane can be calculated as

3 $$\begin{aligned} E_{t}(r_{t},\phi _{t}, z_{t})= & {} \frac{1}{2\pi }\int _{0}^{\infty }\int _{0}^{2\pi } \mho _{t} (k_{x},k_{y}){\tilde{E}}_{i}(k_{i}, \varphi _{i})\exp [i k_{t}r_{t}\cos (\phi _{t}-\varphi _{t})]\nonumber \\&\times \exp [i k_{tz} z_{t}]k_{t}dk_{t}d\varphi _{t} , \end{aligned}$$

where, $\mho _{t} (k_{x},k_{y})$ is the total transmission coefficient, $k_x=k_i cos(\theta _i)+\sqrt{k_{i}^{2}-k_{ix}^{2}-k_{iy}^{2}}\sin (\theta _{i})$, $k_y=k_{iy}$, and $k_{\alpha z}=\sqrt{k_{0}^{2}-k_{\alpha x}^{2}-k_{\alpha y}^{2}}$.

### Negative refraction index

It is well established that employing an external magnetic field can induce strong anisotropy in the ferrite and plasma layers^13^. Due to this fact, the magnetic permeability tensor of the ferrite layer can be described as

4 $$\begin{aligned} {\overline{\mu }}_{F}= \left[ \begin{array}{ccc} \mu _{1} &{} i\mu _{2} &{} 0 \\ -i\mu _{2} &{} \mu _{1}&{} 0 \\ 0 &{} 0 &{} \mu _{3} \\ \end{array} \right] , \end{aligned}$$

in which, $\mu _{1}=1+\omega _{m} (\omega _{0}+i\sigma \omega )/[(\omega _{0}+i\sigma \omega )^2-\omega ^2]$, $\mu _{2}=\omega _{m} \omega /[(\omega _{0}+i\sigma \omega )^2-\omega ^2]$, and $\mu _{3}=\mu _{0}$ with $\omega _{m}=2\pi \gamma M_{0}$ being the characteristic circular frequency, $\omega _{0}=2\pi \gamma H_{0}$ being the resonance frequency, and $\mu _{0}$ being the magnetic permeability of the vacuum. Also, $\gamma =2.8$ *MHz*/*Oe* is the gyromagnetic ratio, $M_{0}$ is the saturation magnetization, $H_{0}$ is the static magnetic field, $\sigma$ is the damping rate. On another side, the electric permittivity tensor of plasma layer under the external magnetization becomes

5 $$\begin{aligned} {\overline{\varepsilon }}_{P}= \left[ \begin{array}{ccc} \varepsilon _{1} &{} i\varepsilon _{2} &{} 0 \\ -i\varepsilon _{2} &{} \varepsilon _{1}&{} 0 \\ 0 &{} 0 &{} \varepsilon _{3} \\ \end{array} \right] , \end{aligned}$$

where, $\varepsilon _{1}=1-\omega _{p}^2 (\omega +i\vartheta )/\omega [(\omega +i\vartheta )^2-\omega _{c}^2]$, $\varepsilon _{2}=-\omega _{p}^2 \omega _{c}/\omega [(\omega +i\vartheta )^2-\omega _{c}^2]$, and $\varepsilon _{3}=1-\omega _{p}^2/\omega (\omega +i\vartheta )$, with $\omega _{p}=\sqrt{4\pi n_{e}e^{2}/m_{e}}$ being the plasma electron frequency, $\omega _{c}=eH_{0}/cm_{e}$ being the cyclotron frequency of electron, and $\vartheta$ is the collisional frequency.

Let us suppose that the wavelength ($\lambda$) of the incoming VB is much greater than the period length ($d=d_{F}+d_{P}$) of the structure. Within this limit, our stratified structure can be regarded as a homogeneous medium, and the averaged permeability ${\overline{\mu }}$ and permittivity ${\overline{\varepsilon }}$ tensors of such system are given by^45^

6 $$\begin{aligned} {\overline{\chi }}= \left[ \begin{array}{ccc} \frac{1}{d}(\chi _{1,m}d_{m}+\chi _{n}d_{n}) &{} \frac{i}{d}(\chi _{2,m}d_{m}) &{} 0 \\ -\frac{i}{d}(\chi _{2,m}d_{m})&{} \frac{1}{d}(\chi _{1,m}d_{m}+\chi _{n}d_{n})&{} 0 \\ 0 &{} 0 &{} [\frac{1}{d}(\frac{d_{m}}{\chi _{3,m}}+\frac{d_{n}}{\chi _{n}})]^{-1} \\ \end{array} \right] , \end{aligned}$$

In the above relation, $\chi \in \{\mu , \varepsilon \}$, and $m,n \in \{P,F\}$. It should be noted that the subscripts *P* and *F* refer to the plasma and Ferrite layers, respectively. To ensure that the considered structure acts as a metamaterial, it is necessary to obtain it’s effective refraction index. According to the Maxwell equations, the electric field inside the plasma-ferrite unit cell satisfies the following relation

7 $$\begin{aligned} \vec {k}\times [{\bar{\mu }}^{(-1)}.(\vec {k}\times \vec {E})]+\frac{\omega ^{2}}{c^{2}}{\overline{\varepsilon }}.\vec {E}=0. \end{aligned}$$

Equation (7) can be simplified into the matrix form

8 $$\begin{aligned} \left[ \begin{array}{ccc} -N^{2}\frac{\mu _{11}}{\rho }\cos ^{2}\delta +\varepsilon _{11} &{} N^{2}\frac{\mu _{12}}{\rho }\cos ^{2}\delta +\varepsilon _{12} &{} N^{2}\frac{\mu _{11}}{\rho }\sin \delta .\cos \delta \\ N^{2}\frac{\mu _{21}}{\rho }\cos ^{2}\delta +\varepsilon _{21}&{} -N^{2}\frac{\mu _{22}}{\rho }\cos ^{2}\delta -N^{2}\sin ^{2}\delta +\varepsilon _{22}&{} N^{2}\frac{\mu _{12}}{\rho }\sin \delta .\cos \delta \\ N^{2}\frac{\mu _{22}}{\rho }\sin \delta .\cos \delta &{} N^{2}\frac{\mu _{21}}{\rho }\sin \delta .\cos \delta &{} -N^{2}\frac{\mu _{11}}{\rho }\sin ^{2}\delta +\varepsilon _{33} \\ \end{array} \right] . \left[ \begin{array}{c} E_{x}\\ E_{y}\\ E_{z}\\ \end{array} \right] =0 , \end{aligned}$$

where, $N=ck/\omega$ is the effective refraction index, $\rho =\mu _{11}^{2}+\mu _{12}^{2}$, and $\delta$ is the angle between the external magnetic field $\vec {H}_{0}$ and wave vector $\vec {k}$. Also, $\mu _{11}$, $\mu _{12}$, $\mu _{21}$, and $\mu _{22}$ are the elements of ${\bar{\mu }}$, and $\varepsilon _{11}$, $\varepsilon _{12}$, $\varepsilon _{21}$, $\varepsilon _{22}$, and $\varepsilon _{33}$ are the elements of ${\bar{\varepsilon }}$, which are extracted from Eq. (6). For a special case, when the incoming VB propagates along the external magnetic field ($\delta =0$), the nontrivial solutions of the Eq. (8) can be given by

9 $$\begin{aligned} N_{\pm }^{2}=(\varepsilon _{11}\pm i\varepsilon _{21})(\mu _{11}\pm i\mu _{21}). \end{aligned}$$

Here, $\varepsilon _{eff}=\varepsilon _{11}\pm i\varepsilon _{21}$ is the effective electric permittivity, and $\mu _{eff}=\mu _{11}\pm i\mu _{21}$ is the effective magnetic permeability. Moreover, the subscripts “$+$” and “−” relate to the ordinary and extraordinary modes, respectively. It should be pointed out that when a homogeneous medium has simultaneously negative permittivity and permeability ($\varepsilon _{eff}<0, \mu _{eff}<0$), one can find some ranges of frequency at which effective refraction of index remains positive and, consequently the wave is allowed to propagate. It can be simply shown that in the case of extraordinary modes in Eq. (9), $\mu _{eff}$ cannot be negative. Thereby, the ordinary modes are the only candidates for having negative refraction index, which will be discussed in the next section.

### Transfer matrix formalism

It is well established that $4\times 4$ transfer matrix approach can accurately describe the optical field propagation inside the complex materials^40^. In this method, after combining the matrix form of Maxwell equations and omitting the longitudinal parts of electromagnetic fields, the wave equation in each anisotropic layer can be written in the compact form

10 $$\begin{aligned} i k_{0}{\bar{\Delta }}\vec {\eta }=\frac{\partial }{\partial z}\vec {\eta }, \end{aligned}$$

in which $k_0=\omega / c$, $$\vec {\eta }= \left[ \begin{array}{cccc} \sqrt{\varepsilon _{0}} E_{x}(z)&\sqrt{\mu _{0}} H_{y}(z)&\sqrt{\varepsilon _{0}} E_{y}(z)&\sqrt{\mu _{0}} H_{x}(z) \end{array} \right] ^{T}$$, and ${\bar{\Delta }}$ is characteristic the matrix or coefficient matrix obtained for the plasma-ferrite unit cell as

11 $$\begin{aligned} {\bar{\Delta }}= \left[ \begin{array}{cccc} 0 &{} ({\tilde{\mu }}_{22}-\frac{{\tilde{k}}_{x}^{2}}{{\tilde{\varepsilon }}_{33}}) &{} 0 &{} (\frac{{\tilde{k}}_{x} {\tilde{k}}_{y}}{{\tilde{\varepsilon }}_{33}}+{\tilde{\mu }}_{21})\\ ({\tilde{\varepsilon }}_{11}-\frac{{\tilde{k}}_{y}^{2}}{{\tilde{\mu }}_{33}}) &{} 0 &{} (\frac{{\tilde{k}}_{x} {\tilde{k}}_{y}}{{\tilde{\mu }}_{33}}+ {\tilde{\varepsilon }}_{12})&{} 0 \\ 0 &{} -(\frac{{\tilde{k}}_{x} {\tilde{k}}_{y}}{{\tilde{\varepsilon }}_{33}}+{\tilde{\mu }}_{12}) &{} 0 &{} (\frac{{\tilde{k}}_{y}^{2}}{{\tilde{\varepsilon }}_{33}}-{\tilde{\mu }}_{11}) \\ -(\frac{{\tilde{k}}_{x} {\tilde{k}}_{y}}{{\tilde{\mu }}_{33}}+ {\tilde{\varepsilon }}_{21}) &{} 0 &{}(\frac{{\tilde{k}}_{x}^{2}}{{\tilde{\mu }}_{33}}-{\tilde{\varepsilon }}_{22}) &{} 0 \end{array} \right] , \end{aligned}$$

where, ${\tilde{k}}_{x}=k_{x}/k_{0}$ , ${\tilde{k}}_{y}=k_{y}/k_{0}$ , ${\tilde{\varepsilon }}_{j\acute{j}}={\varepsilon }_{j\acute{j}}/{\varepsilon }_{0}$ , and ${\tilde{\mu }}_{j\acute{j}}=\frac{\mu _{jj}}{\mu _{0}}$ with $j,\acute{j}=\{1,2,3\}$.

There are four possible periodic solutions for Eq. (10), which are represented in the form

12 $$\begin{aligned} \vec {\eta }_{\xi }(z)=\vec {\eta }_{\xi }(0)\exp [iq_{\xi }z], \end{aligned}$$

where, $\vec {\eta }_{\xi }(0)$ is the value of $\vec {\eta }$ at the entrance face of the considered layer, $q_{\xi }$ is the longitudinal component of the wave vector, and $\xi =\{1,2,3,4\}$. Solving Eq. (10) with the help of eigenmodes introduced by Eq. (12), the transfer matrix of the transverse electric and magnetic field components in each layer becomes

13 $$\begin{aligned} {\bar{G}}(d)={\bar{\Psi }}{\bar{Q}}(d){\bar{\Psi }}^{-1}, \end{aligned}$$

in which *d* is the thickness of each unit cell, ${\bar{Q}}(d)$ is a $4\times 4$ diagonal matrix whose elements are $exp[iq_{\xi }d]$, and ${\bar{\Psi }}$ is a $4\times 4$ matrix composed of $k_{0}{\bar{\Delta }}$ eigenvectors. The transfer matrix for a multilayer structure is obtained via chain multiplication as

14 $$\begin{aligned} \Omega _{t}=\Lambda _{t}^{-1} [\prod ^{1}_{n=N}{\bar{G}}_{n}(d)] \Lambda _{i} \Omega _{i} \equiv \bar{\mathscr {T}} \Omega _{i}, \end{aligned}$$

where, $$\Omega _{i}= \left[ \begin{array}{cccc} E_{i}^{\mathscr {P}}&E_{r}^{\mathscr {P}}&E_{i}^{\mathscr {S}}&E_{r}^{\mathscr {S}} \end{array} \right] ^{T}$$, $$\Omega _{t}= \left[ \begin{array}{cccc} E_{t}^{\mathscr {P}}&0&E_{t}^{\mathscr {S}}&0 \end{array} \right] ^{T}$$, $\bar{\mathscr {T}}$ is the total transfer matrix, and $\Lambda _{\beta }$ with $\beta \in \{i,t\}$ is named dynamical matrix which is derived from boundary conditions as

15 $$\begin{aligned} \Lambda _{\beta }= \left[ \begin{array}{cccc} \cos (\theta _{\beta }) &{} -\cos (\theta _{\beta }) &{} 0 &{} 0\\ n_{\beta } &{} n_{\beta } &{} 0 &{} 0 \\ 0 &{} 0 &{} 1 &{} 1 \\ 0 &{} 0 &{} -n_{\beta }\cos (\theta _{\beta }) &{} n_{\beta }\cos (\theta _{\beta }) \end{array} \right] . \end{aligned}$$

It should be stated that $n_{\beta }$ refers to the refraction index of entrance and exit regions, and $\mathscr {P}$ and $\mathscr {S}$ denote the polarization of the electric field. Now, using the total transfer matrix elements, the total transmission coefficient of passed $\mathscr {S}$- and $\mathscr {P}$-polarized VB from magnetized PFMM are extracted as follows

16 $$\begin{aligned} \mho _{t}^{\mathscr {SS}}= & {} \frac{\mathscr {T}_{11}}{\mathscr {T}_{33}\mathscr {T}_{11}-\mathscr {T}_{13}\mathscr {T}_{31}}, \end{aligned}$$

17 $$\begin{aligned} \mho _{t}^{\mathscr {PP}}= & {} \frac{\mathscr {T}_{33}}{\mathscr {T}_{33}\mathscr {T}_{11}-\mathscr {T}_{13}\mathscr {T}_{31}}, \end{aligned}$$

18 $$\begin{aligned} \mho _{t}^{\mathscr {SP}}= & {} \frac{-\mathscr {T}_{13}}{\mathscr {T}_{33}\mathscr {T}_{11}-\mathscr {T}_{13}\mathscr {T}_{31}}, \end{aligned}$$

19 $$\begin{aligned} \mho _{t}^{\mathscr {PS}}= & {} \frac{-\mathscr {T}_{31}}{\mathscr {T}_{33}\mathscr {T}_{11}-\mathscr {T}_{13}\mathscr {T}_{31}}. \end{aligned}$$

where, $\mathscr {T}_{\kappa \nu }$ are the total transfer matrix components with $\kappa , \nu =\{1,3\}$.

To obtain the OAM spectrum of the output beam, here we calculate the projection of the electric field into the spiral harmonics $exp[i \zeta \phi ]$ by

20 $$\begin{aligned} E(r, \phi , z)=\frac{1}{\sqrt{2\pi }}\sum ^{+\infty }_{\zeta =-\infty } \psi _{\zeta }(r,z)\exp (i \zeta \phi ), \end{aligned}$$

in which, $\psi _{\zeta }(r,z)=\int ^{2\pi }_{0}E(r, \phi , z)\exp (-i \zeta \phi )d\phi$. The carried energy via each mode can be given by $\mathscr {W}_{\zeta }=\int ^{\infty }_{0}\mid \psi _{\zeta }(r,z)\mid ^{2} r dr$. Therefore, one can infer that the weight of each OAM state can be obtained by

21 $$\begin{aligned} \mathscr {P}_{\zeta }=\frac{\mathscr {W}_{\zeta }}{\sum ^{+\infty }_{\zeta =-\infty } \mathscr {W}_{\zeta }}. \end{aligned}$$

Eventually, employing the total transmission coefficients, the Faraday rotation angle (FRA) of the transmitted beam can be calculated by^13^

22 $$\begin{aligned} \delta _{F}=\frac{1}{2}tan^{-1}[\frac{2Re(\mho _{t}^{\mathscr {SP}}/\mho _{t}^{\mathscr {PP}})}{1-|\mho _{t}^{\mathscr {SP}}/\mho _{t}^{\mathscr {PP}}|^{2}}]. \end{aligned}$$

In the following section, we proceed with some numerical simulations to identify the behavior of LG beam interacting with a magnetized PFMM structure.

## Results and discussion

To carry out the numerical analysis, we consider a stratified structure with *N* unit cells which each of them containing two layers. For plasma layer, we let $d_{P}=0.05$ *cm*, and $\vartheta =0.01$ *GHz*. Also, Yttrium Iron Garnet (YIG) as a ferromagnetic layer is characterized by $d_{F}=0.05$ *cm*, $M_{0}=1750$ *Oe*, $\varepsilon _{F}=25$, and $\alpha =0.002$^46^. It is noted that according to the supposed conditions in the theoretical section, it is necessary to consider the incoming VB wavelength $(\lambda )$ much greater than the period length of the PFMM $(d=d_{F}+d_{P})$. Moreover, the beam waist radius and incident angle of the incoming VB is assumed $R_{0}=6\lambda$, and $\theta _{i}=0$, respectively.

**Figure 2:**
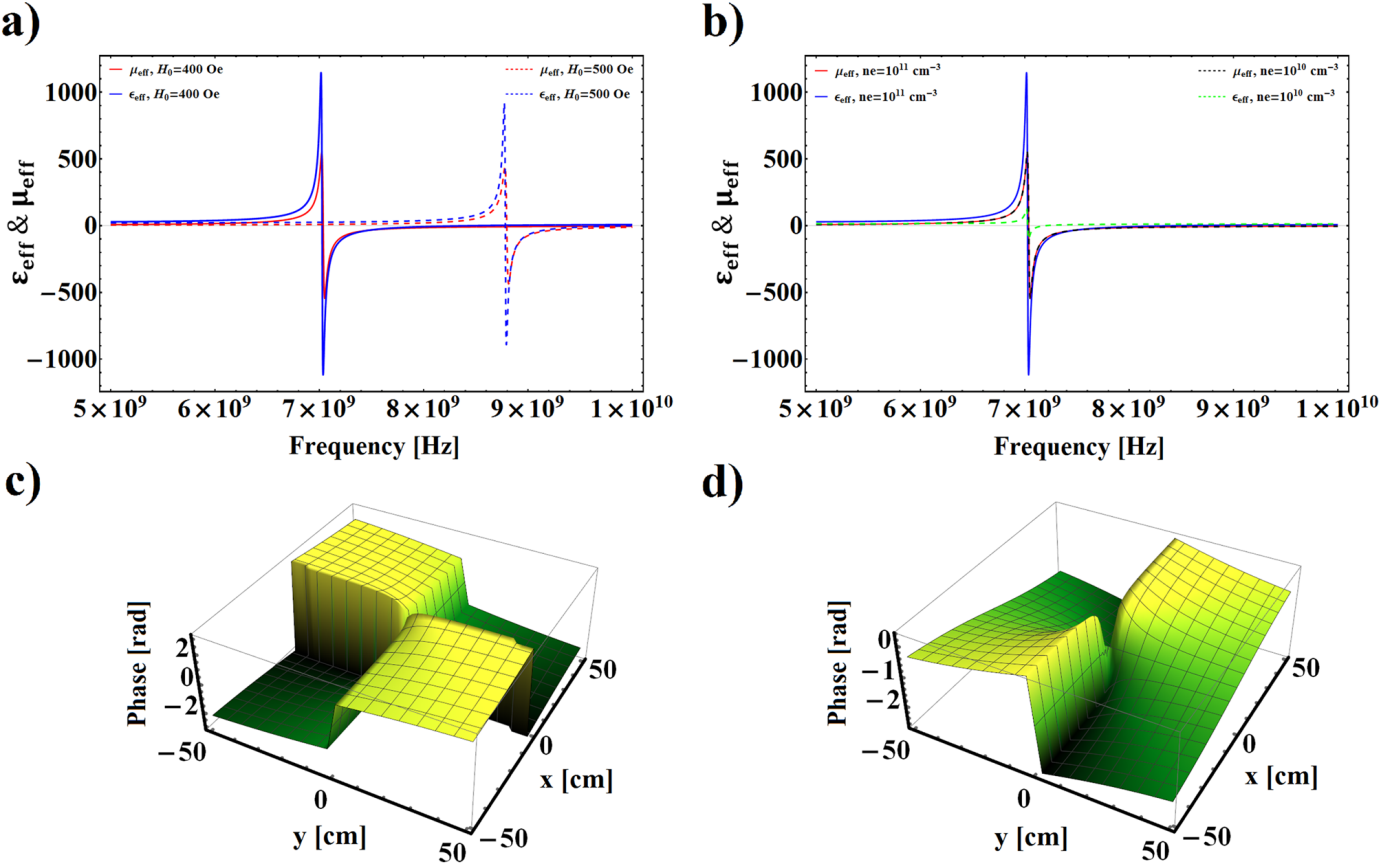
**(a,b)** The real part of effective electric permittivity and magnetic permeability as a function of incoming wave frequency for different values of external electric field and plasma number density, respectively. **(c,d)** Phase distributions of transmitted VB for $\omega _{1}=7.15$ *GHz* and $\omega _{2}=6.82$ *GHz*, respectively, with $H_{0}=400$ *Oe*, $n_{e}=10^{10}$ $cm^{-3}$, $\ell =2$, and $p=0$.

**Figure 3:**
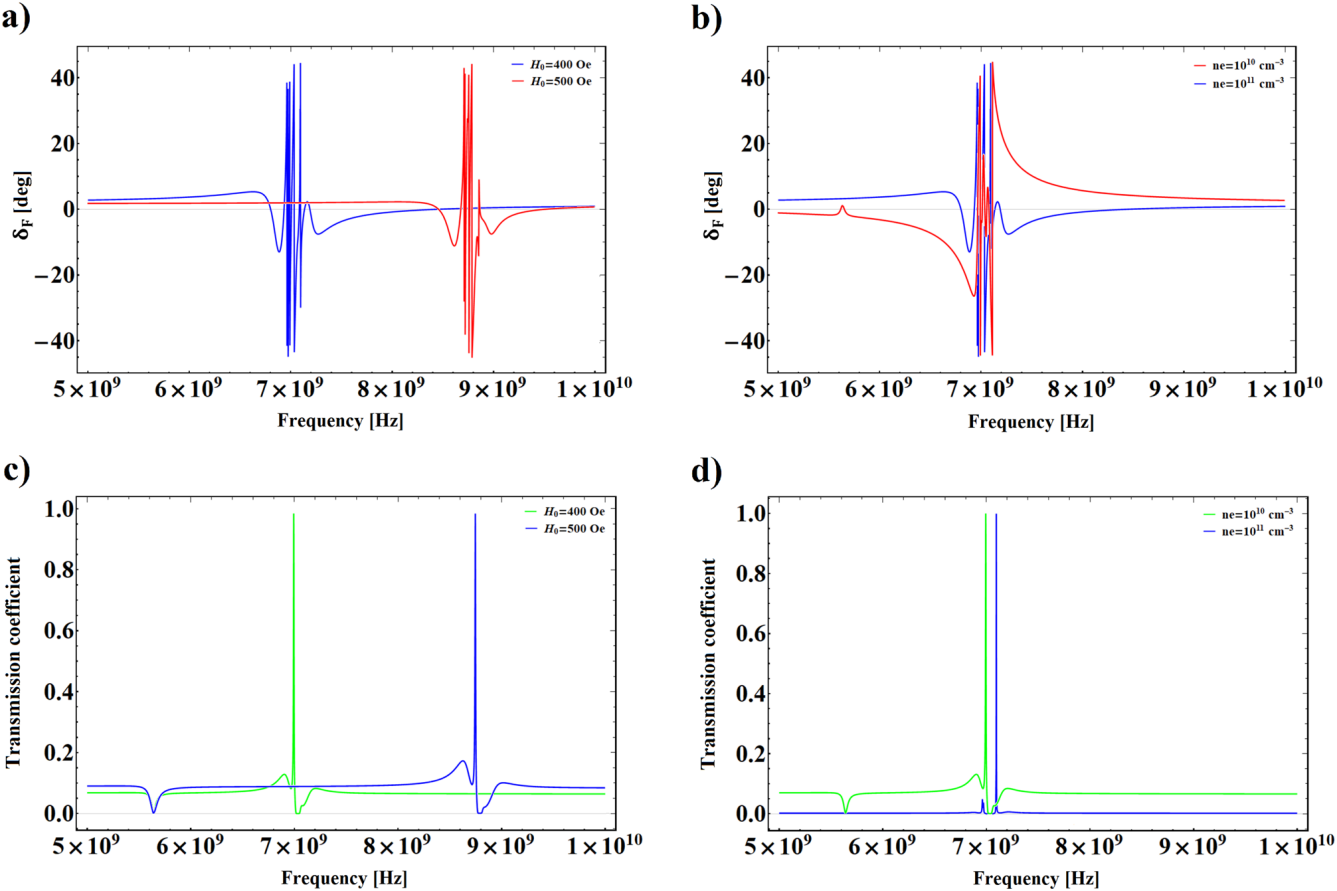
**(a,b)** FRA of transmitted VB beam versus incident wave frequency for different values of external electric field and plasma number density, respectively. **(c,d)** The total transmission coefficient as a function of incident wave frequency for different values of external electric field and plasma number density, respectively.

**Figure 4:**
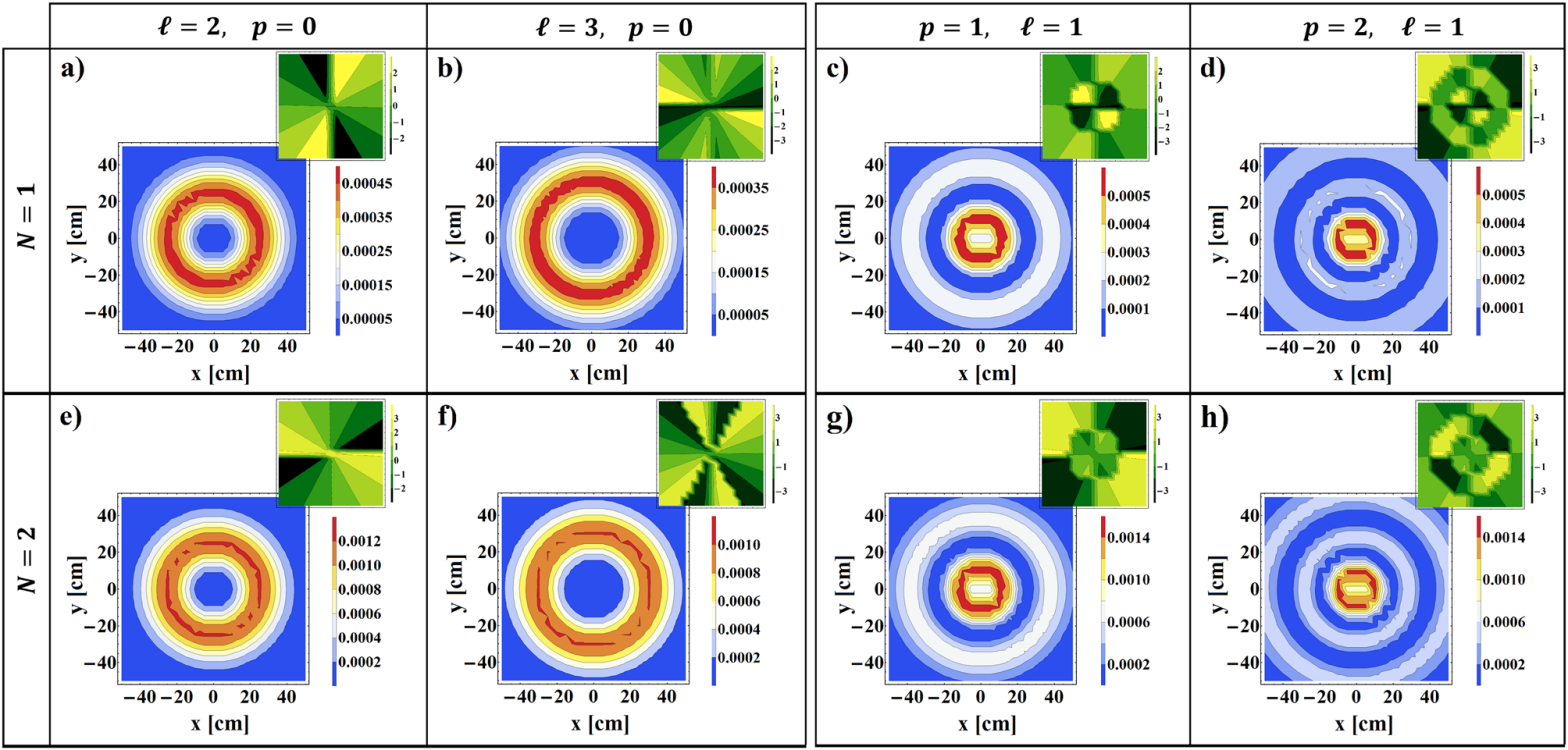
Transverse intensity and corresponding phase distributions of transmitted VB with $n_{e}=10^{11}$ $cm^{-3}$, $H_{0}=400$ *Oe*, $\omega =7.15$ *GHz*, and different values of angular and radial mode numbers for $N=1$ (upper row), and $N=2$ (lower row).

**Figure 5:**
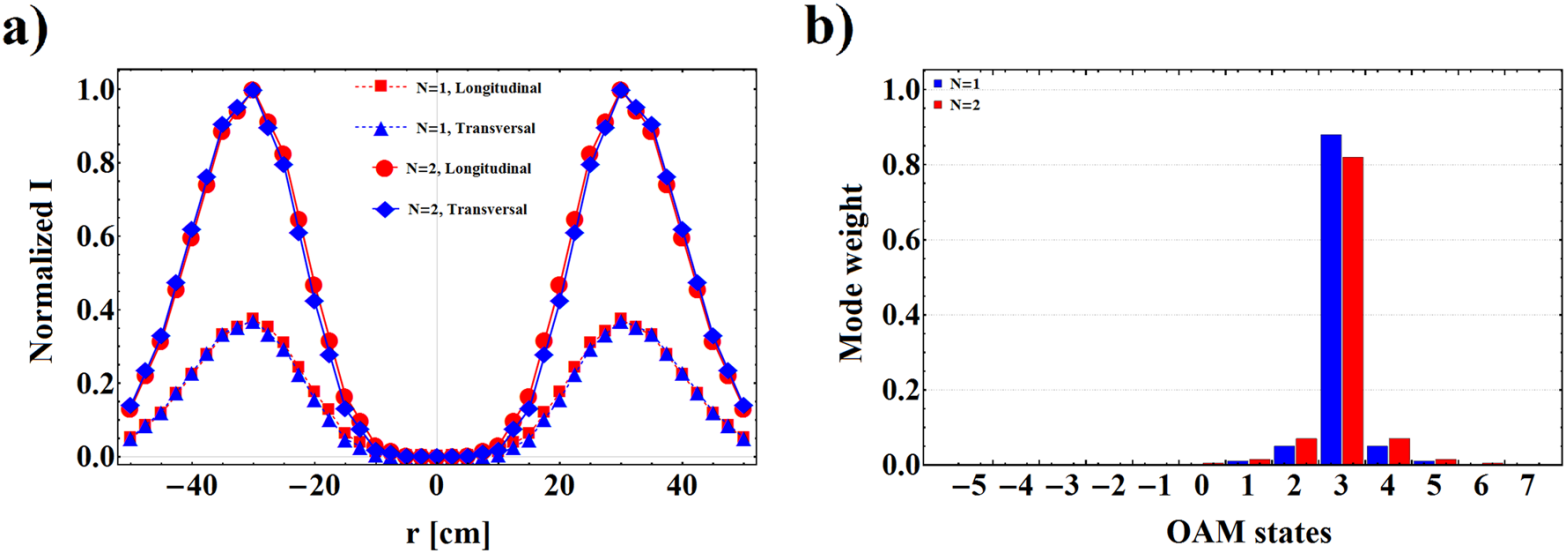
**(a)** Comparison of normalized transversal and longitudinal intensity distribution of transmitted VB for $N=1$ and $N=2$, with $n_{e}=10^{11}$ $cm^{-3}$, $H_{0}=400$ *Oe*, $\ell =3$, $p=0$, and $\omega =7.15$ *GHz*. **(b)** Comparison of transmitted topological charge mode weight for $N=1$ and $N=2$, with the other parameters same as part **(a)**.

Figure 2a,b show the variation of effective electric permittivity $(\varepsilon _{eff})$ and magnetic permeability $(\mu _{eff})$ of plasma-ferrite composite as a function of incident VB frequency $(\omega )$, with different values of external magnetic field and plasma number density, respectively. In Fig. 2a, which is plotted for a fixed value of plasma number density $n_{e}=10^{11}$ cm$^{-3}$, it can be seen that for $H_{0}=400$ *Oe*, within the frequency domain over 7 *GHz* both the $\varepsilon _{eff}$ and $\mu _{eff}$ have negative values, and the negative refraction index condition is fulfilled in this frequency range. By increasing $H_{0}$ up to $H_{0}=500$ *Oe* the frequency range related to the negative refraction index shifts toward higher values. To show the tunability of the $\varepsilon _{eff}$ and $\mu _{eff}$ with variation of plasma number density, Fig. 2b is plotted for fixed values of $H_{0}=400$ *Oe*, and two different values of $n_{e}$. It is clear that increasing plasma number density from $n_{e}=10^{10}$ cm$^{-3}$ to $n_{e}=10^{11}$ cm$^{-3}$ does not lead to a considerable effect on the displacement of the resonance frequency, but cusses to increase the value of $\varepsilon _{eff}$ in the resonance region.

Figure  2c,d illustrate the influence of the plasma-ferrite structure on the phase distribution of the incoming VB in the left-handed and right-handed frequency domains, respectively, with $H_{0}=400$ *Oe*, $n_{e}=10^{10}$ cm$^{-3}$, $\ell =2$, $p=0$, and two different values of incident frequencies $\omega _{1}=7.15$ GHz and $\omega _{2}=6.82$ GHz. By comparing these two phase profiles of transmitted VB it is concluded that in the incident frequency which the structure displays negative refraction index the transmitted phase front rotates clockwise, while in the other incident frequency associated to the positive refraction index it rotates anti-clockwise. This reversed rotation of the wave front of the VB in the left-handed materials arises from negative phase velocity $(v_{ph}=\omega /k)$ of the vortex field during propagation in metamaterials. As well as, with a precise look at the Fig. 2c,d it is inferred that the optical angular momentum of the transmitted VB can be modulated through variation of the incoming wave frequency. This phenomenon may be related to the dislocation of the output beam with incoming wave frequency.

In Fig. 3a,b we depict the evolution of FRA versus the incident VB frequency for different values of external magnetic field and plasma number density. It is obvious from these figures that FRA changes suddenly in the same wave frequency range which the system exhibits left-handed behavior, as well as, due to the increment of difference between effective refraction index of left and right circularly polarized modes of propagating wave, a large amount of Faraday rotation emerges in those frequencies. In the Fig. 3a, where $n_{e}=10^{11}$ cm$^{-3}$, it is observed that the maximum value of $\delta _{F}$ is sensitive to the variation of $H_{0}$, and as $H_{0}$ increases the maximum value of $\delta _{F}$ remarkably moves toward higher frequencies. The dependence of FRA on the variation of plasma number density is illustrated in Fig. 3b for $H_{0}=400$ *Oe*, and two different values of $n_{e}$. As shown in this figure, in the non-resonance frequency region decreasing the plasma number density gives rise to increasing $\delta _{F}$, and its maximum value in the resonance region is not very sensitive to the variation of $n_{e}$. Such increasing of $\delta _{F}$ with decreasing $n_{e}$ can be attributed to the transferability of the structure. To show the trend of energy transmission from the structure in corresponding frequency limit, Fig. 3c,d have been plotted for different values of $H_{0}$ and $n_{e}$. It is shown that there is a significant amount of energy transmission in the vicinity of the resonance region. In general, multilayered structures in which negative refraction index materials are included can be considered as a perfect lens and, therefore, they are envisaged to possess unique transmission features^4^. On the other hand, this figures show that increasing the external magnetic field or plasma number density causes to shift the maximum value of pass-band toward the higher frequency region. It should be noted that, the effect of magnetic field increment is stronger than that of the plasma number density in the displacement of the pass-band maximum. These controllable properties of plasma-ferrite structure can be of great importance in engineering applications.

**Figure 6:**
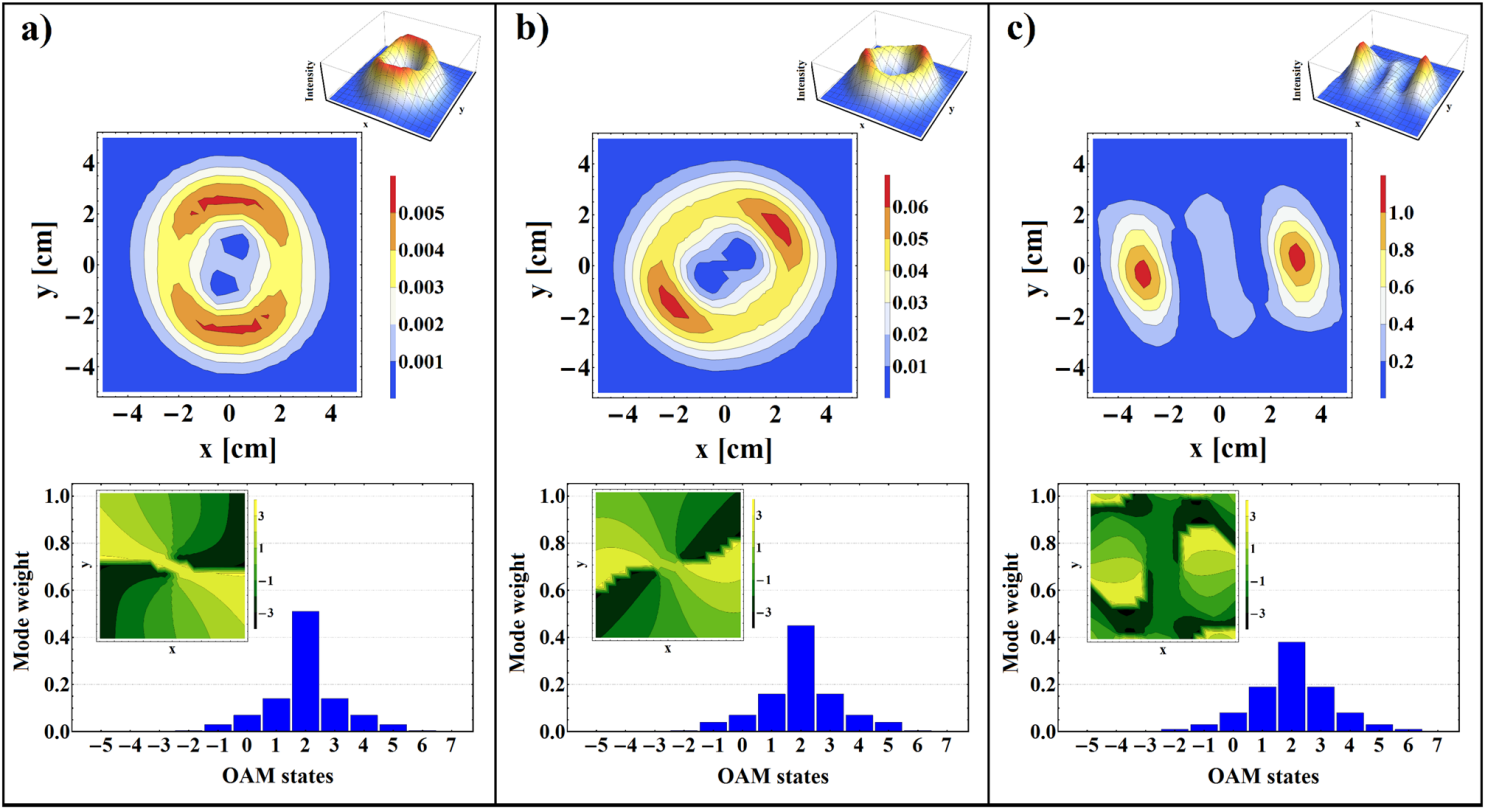
Intensity profiles accompanied by corresponding phase maps and OAM spectrums of transmitted VB for $H_{0}=4000$ *Oe*, $\ell =2$, $p=0$, $\omega =71.5$ GHz, and **(a)** $n_{e}=2.5\times 10^{12}$ cm$^{-3}$, **(b)** $n_{e}=3.5\times 10^{12}$ cm$^{-3}$, (c) $n_{e}=4.5\times 10^{12}$ cm$^{-3}$.

The intensity profiles beside the corresponding phase maps of the transmitted VB from plasma-ferrite composites with $N=1$, and $N=2$ are represented in Fig. 4 for $n_{e}=10^{11}$ cm$^{-3}$, $H_{0}=400$ *Oe*, $\omega =7.15$ GHz, and several quantities of angular and radial mode numbers. It seems that the transmitted intensity profiles for both quantities of unit cells maintain their circular shape, and their corresponding phase distributions exhibit screw like form. Nevertheless, presence of some distortion in the intensity profiles of the output beam is undeniable so that enhancing the number of unit cells increases the amount of those perturbations. Such phenomenon is attributed to the emergence of the sideband modes of incoming VB during the propagation in the stratified medium^47,48^. In fact, throughout the photonic tunneling the incoming vortex wave is perturbed, and new vortex modes are born and superposed together. It is well proved that this phenomenon is associated with some geometrical behaviors of transmitted beam through stratified media such as Goos-Hänchen and Imbert-Fedorov shifts^49^. These intrinsic features in some cases lead to giant reconstruction of the intensity and phase profiles of the passing beam^50,51^, and have potential application in data encoding for wireless communications, switching, beam shaping, and other OAM-based applications^52^. The existence of these perturbations in the output beam can be confirmed through phase distributions. It is well known that phase maps give a convenient and precise way to diagnosis the angular and radial indices of the beam. Indeed, appearance the phase leap from zero to $2\ell \pi$ around the singular point, and emergence a number of $p\pi$-shift boundary rings from radial direction, identify the values of angular and radial indices, respectively. A closer look at the phase maps of the output beam indicates some deformations or additional portions in their distributions, which verify the generation and superposition of sideband modes in the output patterns. For further evaluation, in Fig. 5a,b we compare the normalized intensity distributions and OAM spectrums of the output beam for $N=1$ and $N=2$, respectively. For this comparison we let $n_{e}=10^{11}$ $cm^{-3}$, $H_{0}=400$ *Oe*, $\ell =3$, $p=0$, and $\omega =7.15$ *GHz*. From Fig. 5a it is evident that interference of neighboring modes in the output plane not only deforms the shape of output beams but also generates an asymmetry in their intensity distributions. Fig. 5b demonstrates that for both numbers of unit cells the weight or purity of primary topological charge decreases. In addition, reducing the OAM mode purity increases with increasing the unit cell number.

The tunability of the intensity profiles, phase maps, and spiral spectrums of the transmitted VB with plasma number density is presented in Fig. 6 for $H_{0}=4000$ *Oe*, $\ell =2$, $p=0$, $\omega =71.5$ GHz, and $n_{e}=2.5\times 10^{12}$ cm$^{-3}$, $n_{e}=3.5\times 10^{12}$ cm$^{-3}$, and $n_{e}=4.5\times 10^{12}$ cm$^{-3}$. As shown in this figure, for higher value of external magnetic field and within the negative refraction index frequency range by enhancing the plasma number density reshaping of the transmitted beam occurs, so that, while the magnitude of $n_{e}$ reaches to $4.5\times 10^{12}$ cm$^{-3}$ two distinct intensity bumps become visible in the output plane. These intensity variations are in good agreement with 3*D* intensity plots and phase maps which plainly exhibit the reshaping and reconstruction of transmitted beam under the influence of variable parameters. Furthermore, to explore the reason of such significant reshaping, we get help from OAM spectrum of transmitted beams shown in the bottom row of the Fig. 6. It is apparent that for higher values of the plasma number density sideband modes are generated with more weight, and the purity of primary topological charge gradually decreases. Apart from the reshaping of the output patterns, one can see a rotation of the twin part of intensity profile with increasing $n_{e}$. As is known, this shape rotation is originated from circular birefringence of plasma-ferrite layer in the presence of external magnetic field^14,53,54^, and can be adjusted via variation of the plasma number density and other external parameters.

**Figure 7:**
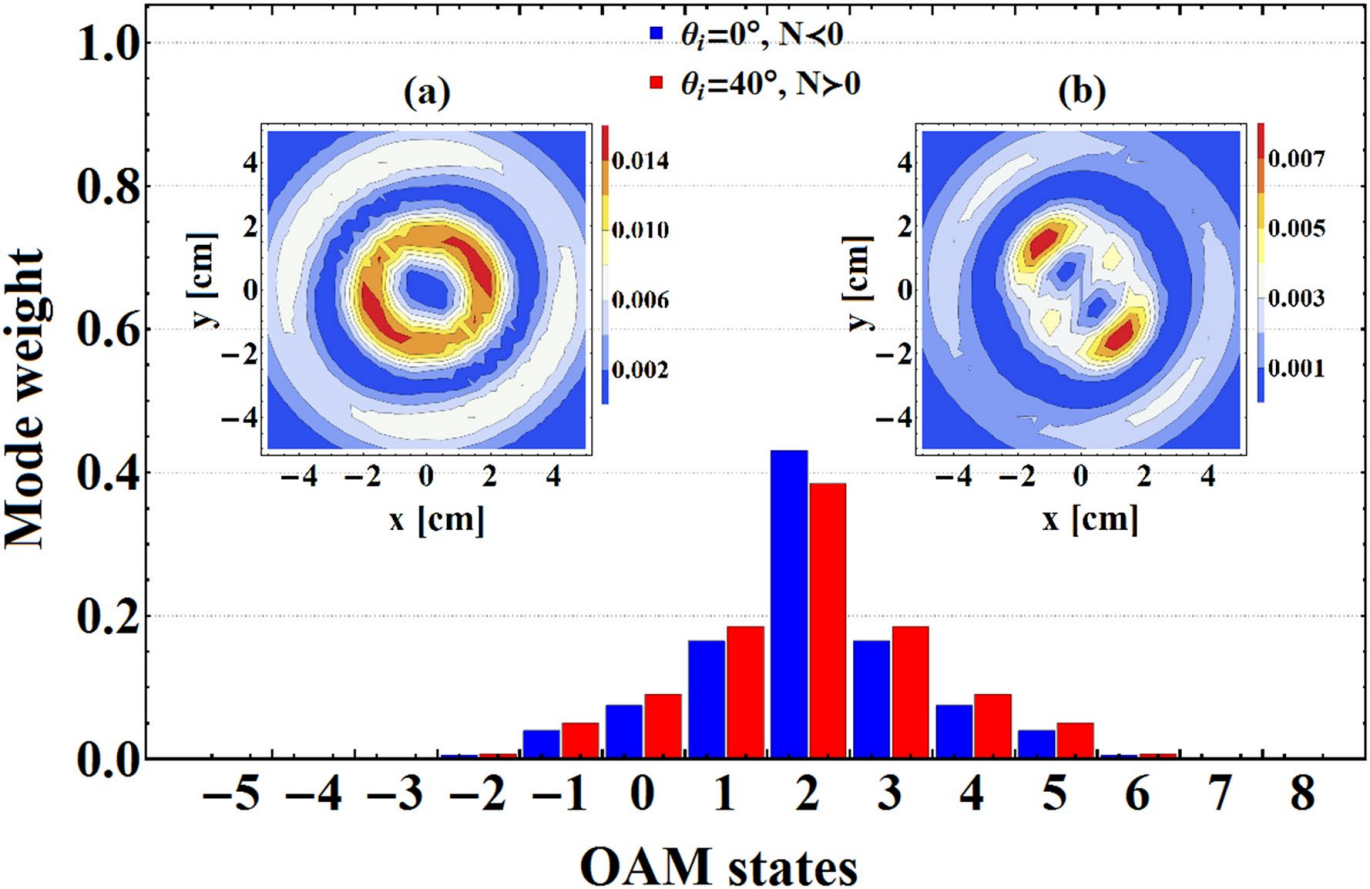
OAM spectrums of transmitted VB for $H_{0}=4000$ *Oe*, $\ell =2$, $p=1$, $\omega =71.5$ GHz, and $n_{e}=3\times 10^{12}$ cm$^{-3}$. The insets represent intensity distributions of output VB in **(a)** normal and **(b)** oblique incidence.

Eventually, in Fig. 7 we provide a comparison between mode purity of the output beams in normal and oblique incidence for $\ell =2$, $p=1$, $H_{0}=4000$ *Oe*, $\omega =71.5$ GHz, and $n_{e}=3\times 10^{12}$ cm$^{-3}$. Also, we represent the intensity profiles of transmitted VB for both normal and oblique incidence in Fig. 7a,b , respectively. From this figure it is plain that for the oblique incidence the weight of primary OAM state in the transmitted beam decreases and the neighboring modes emerge with higher weight. This leads to increase the deformation of the output beam profile of oblique incidence in comparison with the normal incidence ones.

## Conclusions

To summarize, we have theoretically studied the transmission of microwave vortex beams (VB) from a magnetized plasma-ferrite metamaterial (PFMM) by applying the angular spectrum expansion approach accompanied by the $4\times 4$ transfer matrix method. From numerical simulations, it has been identified that plasma-ferrite structures exhibit remarkable pass-band and large Faraday rotation angles in the vicinity of the magnetic resonance frequency. These effects are greatly controlled through some external parameters such as imposed static magnetic field and plasma number density. Furthermore, it has been shown that due to the negative phase velocity, the wave front of VB reversely rotates while propagating through PFMM. The variation of intensity profiles and phase distributions of the transmitted beam with different radial and angular mode numbers has been checked out for different unit cell numbers, and from the comparison of their spiral spectrums it has been found out that throughout the photonic tunneling the incoming VB is perturbed, and novel side-band modes are born and superposed together. Emergence of such side-band modes in the higher values of external magnetic field gives rise to the reshaping and reconstruction of the output beam. We believe that this investigation not only clarifies the behavior of VBs passing through magnetic metamaterials, but also provides a pathway for manipulation of them via tunable stratified structures.
